# Methyl 2-[(methyl­sulfon­yl)(prop­yl)amino]benzoate

**DOI:** 10.1107/S1600536809054920

**Published:** 2009-12-24

**Authors:** Muhammad Shafiq, M. Nawaz Tahir, Islam Ullah Khan, Muhammad Nadeem Arshad, Zeeshan Haider

**Affiliations:** a Department of Chemistry, Government College University, Lahore, Pakistan; b Department of Physics, University of Sargodha, Sargodha, Pakistan

## Abstract

The asymmetric unit of the title compound, C_12_H_17_NO_4_S, contains two mol­ecules, both of which show disorder of the two terminal C atoms of the propyl chain over two sets of sites with an occupancy ratio of 0.581 (6):0.419 (6). Intra­molecular C—H⋯O inter­actions help to establish the mol­ecular conformations: in one mol­ecule, the dihedral angle between the methyl ester group and the benzene ring is 41.0 (2)°, whereas in the other mol­ecule it is 36.12 (17)°. In the crystal, mol­ecules are linked by inter­molecular C—H⋯O and C—H⋯π inter­actions.

## Related literature

For related structures, see: Shafiq *et al.* (2008 ▶, 2009*a* ▶,*b* ▶).
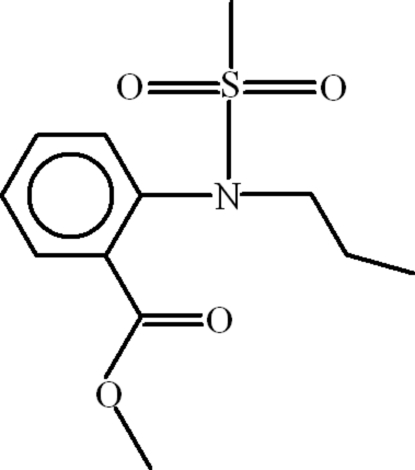


## Experimental

### 

#### Crystal data

C_12_H_17_NO_4_S
*M* *_r_* = 271.33
Monoclinic, 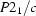

*a* = 10.1762 (4) Å
*b* = 15.0178 (6) Å
*c* = 18.0900 (8) Å
β = 92.787 (2)°
*V* = 2761.3 (2) Å^3^
*Z* = 8
Mo *K*α radiation
μ = 0.24 mm^−1^
*T* = 296 K
0.25 × 0.12 × 0.10 mm

#### Data collection

Bruker Kappa APEXII CCD diffractometer
Absorption correction: multi-scan (*SADABS*; Bruker, 2005 ▶) *T*_min_ = 0.955, *T*_max_ = 0.968
25991 measured reflections
5409 independent reflections
2607 reflections with *I* > 2σ(*I*)
*R*_int_ = 0.078

#### Refinement

*R*[*F* ^2^ > 2σ(*F* ^2^)] = 0.062
*wR*(*F* ^2^) = 0.193
*S* = 1.01
5409 reflections
328 parameters
7 restraints
H-atom parameters constrained
Δρ_max_ = 0.50 e Å^−3^
Δρ_min_ = −0.40 e Å^−3^

### 

Data collection: *APEX2* (Bruker, 2007 ▶); cell refinement: *SAINT* (Bruker, 2007 ▶); data reduction: *SAINT*; program(s) used to solve structure: *SHELXS97* (Sheldrick, 2008 ▶); program(s) used to refine structure: *SHELXL97* (Sheldrick, 2008 ▶); molecular graphics: *ORTEP-3* (Farrugia, 1997 ▶) and *PLATON* (Spek, 2009 ▶); software used to prepare material for publication: *WinGX* (Farrugia, 1999 ▶) and *PLATON*.

## Supplementary Material

Crystal structure: contains datablocks global, I. DOI: 10.1107/S1600536809054920/hb5288sup1.cif

Structure factors: contains datablocks I. DOI: 10.1107/S1600536809054920/hb5288Isup2.hkl

Additional supplementary materials:  crystallographic information; 3D view; checkCIF report

## Figures and Tables

**Table 1:** Hydrogen-bond geometry (Å, °) *Cg*1 and *Cg*2 are the centroids of the C2–C7 and C14–C19 benzene rings, respectively.

*D*—H⋯*A*	*D*—H	H⋯*A*	*D*⋯*A*	*D*—H⋯*A*
C6—H6⋯O8^i^	0.93	2.59	3.483 (6)	161
C11*A*—H11*C*⋯O1	0.96	2.57	3.486 (15)	159
C16—H16⋯O4	0.93	2.51	3.429 (5)	168
C20—H20*C*⋯O3^ii^	0.96	2.57	3.172 (6)	121
C21—H21*A*⋯O1	0.97	2.55	3.192 (6)	124
C12—H12*A*⋯*Cg*2^iii^	0.96	2.84	3.660 (5)	144
C18—H18⋯*Cg*1^iv^	0.93	2.87	3.588 (5)	135

Symmetry codes: (i) 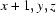; (ii) 
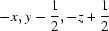
; (iii) 
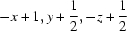
; (iv) 
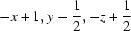
.

**Fig. 1.**
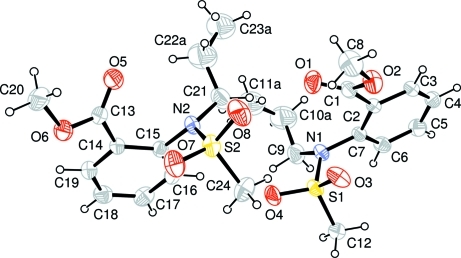
View of (I) with the atom numbering scheme having atoms of greater occupancy ratio. The thermal ellipsoids are drawn at the 30% probability level. H-atoms are shown by small circles of arbitrary radii.

**Fig. 2.**
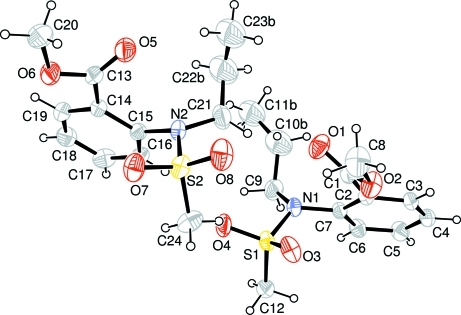
View of (I) with the atom numbering scheme having atoms of smaller occupancy ratio. The thermal ellipsoids are drawn at the 30% probability level. H-atoms are shown by small circles of arbitrary radii.

**Fig. 3.**
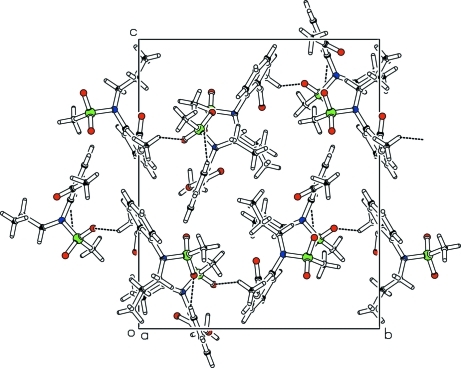
The partial packing of (I), which shows that the molecules are stacked in polymeric chains due to intermolecular H-bodings that extend along the crystallographic b axis.

## supplementary crystallographic information

### Comment

In continuation to our work on the synthesis of benzothiazine derivatives, we have reported various molecules of this series (Shafiq *et al.*, 2008, 2009*a*, 2009*b*). The title compound (I, Fig. 1, Fig. 2) is being reported here in this context.

The asymmtric unit of title compound consists of two molecules. In both molecules the two terminal C-atoms of propyl moiety are disordered over two set of sites with occupancy ratio of 0.581 (6):0.419 (6). The two molecules of asymmetric units differ from each other as the dihedral angle between methyl ester A (O2/C1/O1/C8) with benzene ring B (C2–C7) is 40.96 (21)°, whereas in the other molecule it is 36.12 (17)° between C (O6/C13/O5/C20) and D (C13–C19). The molecules are stabilized due to intra as well as intermolecular and C–H···π interactions (Table 1, Fig. 2).

### Experimental

For the preparation of the title compound, the suspension of hexane-washed sodium hydride (50% in mineral oil) was prepared in dry dimethylformamide (3 ml). A solution of methyl *N*-methylsulfonylanthranilate (70 mg, 0.306 mmol) in dry dimethylformamide (5 ml) was added to the suspension and stirred for 45 min at room temperature. Then, a solution of propyl iodide (156.40 mg, 0.92 mmol) was added to it. The resulting white suspension was stirred for 2.5–3 h, added to ice and pH adjusted at 4–4.5 and kept in freezer. Solid product obtained was filtered and dried to obtain white prisms of title compound.

### Refinement

The two terminal C-atoms of each molecule are disordered over two set of sites. The disordered C-atoms were treated anisotropic with EADP and refined using *DFIX*.

H-atoms were positioned geometrically, with C—H = 0.93, 0.96 and 0.97 Å for aryl, methyl and ethylene H, respectively and constrained to ride on their parent atoms, with *U*_iso_(H) = *xU*_eq_(C), where *x* = 1.2 for aryl and 1.5 for methyl H atoms.

### Crystal data

**Table:**

C_12_H_17_NO_4_S	*F*(000) = 1152
*M**_r_* = 271.33	*D*_x_ = 1.305 Mg m^−^^3^
Monoclinic, *P*2_1_/*c*	Mo *K*α radiation, λ = 0.71073 Å
Hall symbol: -P 2ybc	Cell parameters from 2607 reflections
*a* = 10.1762 (4) Å	θ = 2.0–26.0°
*b* = 15.0178 (6) Å	µ = 0.24 mm^−^^1^
*c* = 18.0900 (8) Å	*T* = 296 K
β = 92.787 (2)°	Prismatic, white
*V* = 2761.3 (2) Å^3^	0.25 × 0.12 × 0.10 mm
*Z* = 8	

### Data collection

**Table:**

Bruker Kappa APEXII CCD diffractometer	5409 independent reflections
Radiation source: fine-focus sealed tube	2607 reflections with *I* > 2σ(*I*)
graphite	*R*_int_ = 0.078
Detector resolution: 7.70 pixels mm^-1^	θ_max_ = 26.0°, θ_min_ = 2.0°
ω scans	*h* = −12→12
Absorption correction: multi-scan (*SADABS*; Bruker, 2005)	*k* = −18→18
*T*_min_ = 0.955, *T*_max_ = 0.968	*l* = −22→22
25991 measured reflections	

### Refinement

**Table:**

Refinement on *F*^2^	Primary atom site location: structure-invariant direct methods
Least-squares matrix: full	Secondary atom site location: difference Fourier map
*R*[*F*^2^ > 2σ(*F*^2^)] = 0.062	Hydrogen site location: inferred from neighbouring sites
*wR*(*F*^2^) = 0.193	H-atom parameters constrained
*S* = 1.01	*w* = 1/[σ^2^(*F*_o_^2^) + (0.0837*P*)^2^ + 1.0657*P*] where *P* = (*F*_o_^2^ + 2*F*_c_^2^)/3
5409 reflections	(Δ/σ)_max_ < 0.001
328 parameters	Δρ_max_ = 0.50 e Å^−^^3^
7 restraints	Δρ_min_ = −0.40 e Å^−^^3^

### Special details

**Table:**

Geometry. Bond distances, angles *etc*. have been calculated using the rounded fractional coordinates. All su's are estimated from the variances of the (full) variance-covariance matrix. The cell e.s.d.'s are taken into account in the estimation of distances, angles and torsion angles
Refinement. Refinement of *F*^2^ against ALL reflections. The weighted *R*-factor *wR* and goodness of fit *S* are based on *F*^2^, conventional *R*-factors *R* are based on *F*, with *F* set to zero for negative *F*^2^. The threshold expression of *F*^2^ > σ(*F*^2^) is used only for calculating *R*-factors(gt) *etc*. and is not relevant to the choice of reflections for refinement. *R*-factors based on *F*^2^ are statistically about twice as large as those based on *F*, and *R*- factors based on ALL data will be even larger.

### Fractional atomic coordinates and isotropic or equivalent isotropic displacement parameters (Å^2^)

**Table:**

	*x*	*y*	*z*	*U*_iso_*/*U*_eq_	Occ. (<1)
S1	0.56276 (10)	0.24818 (8)	0.18894 (6)	0.0513 (4)	
O1	0.3570 (3)	0.1598 (3)	0.0442 (2)	0.0822 (15)	
O2	0.3266 (3)	0.2903 (2)	−0.01117 (18)	0.0740 (13)	
O3	0.4772 (3)	0.3112 (2)	0.15293 (16)	0.0706 (13)	
O4	0.5151 (3)	0.1951 (2)	0.24718 (17)	0.0744 (13)	
N1	0.6139 (3)	0.1809 (2)	0.12571 (18)	0.0499 (11)	
C1	0.4009 (4)	0.2243 (3)	0.0166 (2)	0.0544 (19)	
C2	0.5427 (4)	0.2403 (3)	0.0037 (2)	0.0444 (14)	
C3	0.5756 (4)	0.2752 (3)	−0.0643 (2)	0.0527 (16)	
C4	0.7044 (5)	0.2844 (3)	−0.0819 (3)	0.0598 (17)	
C5	0.8031 (4)	0.2615 (3)	−0.0303 (3)	0.0632 (19)	
C6	0.7722 (4)	0.2291 (3)	0.0375 (3)	0.0557 (16)	
C7	0.6419 (4)	0.2167 (3)	0.0549 (2)	0.0450 (14)	
C8	0.1861 (4)	0.2808 (4)	−0.0041 (3)	0.097 (3)	
C9	0.6872 (5)	0.0997 (3)	0.1507 (3)	0.079 (2)	
C10A	0.6733 (13)	0.0159 (9)	0.1078 (9)	0.132 (3)	0.581 (6)
C11A	0.5345 (13)	−0.0144 (10)	0.1283 (8)	0.132 (3)	0.581 (6)
C12	0.6994 (4)	0.3062 (3)	0.2254 (3)	0.0604 (17)	
C11B	0.616 (2)	−0.0690 (11)	0.1372 (10)	0.132 (3)	0.419 (6)
C10B	0.6124 (19)	0.0243 (11)	0.1107 (12)	0.132 (3)	0.419 (6)
S2	0.06182 (10)	0.20113 (8)	0.25426 (7)	0.0580 (4)	
O5	−0.1063 (3)	−0.0120 (3)	0.2656 (2)	0.0812 (14)	
O6	−0.1371 (3)	−0.0088 (2)	0.38509 (17)	0.0717 (13)	
O7	−0.0179 (3)	0.1979 (2)	0.31664 (18)	0.0755 (11)	
O8	0.0053 (3)	0.2294 (3)	0.1850 (2)	0.0885 (14)	
N2	0.1202 (3)	0.1019 (2)	0.24272 (18)	0.0494 (12)	
C13	−0.0629 (4)	−0.0033 (3)	0.3280 (3)	0.0556 (17)	
C14	0.0786 (4)	0.0076 (3)	0.3503 (2)	0.0449 (16)	
C15	0.1667 (4)	0.0543 (3)	0.3081 (2)	0.0448 (16)	
C16	0.2983 (4)	0.0561 (3)	0.3294 (3)	0.0548 (17)	
C17	0.3441 (4)	0.0123 (3)	0.3916 (3)	0.0661 (19)	
C18	0.2600 (5)	−0.0339 (3)	0.4335 (3)	0.0649 (17)	
C19	0.1271 (4)	−0.0351 (3)	0.4137 (3)	0.0569 (17)	
C20	−0.2745 (4)	−0.0294 (4)	0.3686 (3)	0.089 (2)	
C21	0.1850 (5)	0.0829 (4)	0.1726 (3)	0.082 (2)	
C22A	0.1036 (14)	0.0153 (10)	0.1341 (8)	0.132 (3)	0.581 (6)
C23A	0.1593 (14)	−0.0270 (9)	0.0668 (7)	0.132 (3)	0.581 (6)
C24	0.1951 (5)	0.2699 (3)	0.2772 (3)	0.0776 (19)	
C23B	0.0401 (17)	−0.0269 (13)	0.1068 (10)	0.132 (3)	0.419 (6)
C22B	0.1746 (18)	−0.0121 (14)	0.1428 (12)	0.132 (3)	0.419 (6)
H4	0.72516	0.30591	−0.12807	0.0720*	
H3	0.50905	0.29256	−0.09837	0.0634*	
H8A	0.15863	0.22292	−0.02137	0.1453*	
H8B	0.14059	0.32570	−0.03309	0.1453*	
H8C	0.16584	0.28730	0.04692	0.1453*	
H9A	0.77999	0.11479	0.15369	0.0946*	
H9B	0.66248	0.08683	0.20068	0.0946*	
H10A	0.73989	−0.02731	0.12339	0.1582*	0.581 (6)
H10B	0.67740	0.02645	0.05509	0.1582*	0.581 (6)
H11A	0.53481	−0.02692	0.18035	0.1977*	0.581 (6)
H11B	0.51025	−0.06709	0.10089	0.1977*	0.581 (6)
H11C	0.47223	0.03209	0.11640	0.1977*	0.581 (6)
H12A	0.74009	0.33773	0.18643	0.0906*	
H12B	0.76128	0.26493	0.24797	0.0906*	
H12C	0.67209	0.34778	0.26191	0.0906*	
H5	0.89078	0.26802	−0.04155	0.0756*	
H6	0.83923	0.21524	0.07235	0.0665*	
H10C	0.52060	0.04192	0.10716	0.1582*	0.419 (6)
H10D	0.64197	0.02323	0.06052	0.1582*	0.419 (6)
H11D	0.61294	−0.10870	0.09558	0.1977*	0.419 (6)
H11E	0.54081	−0.07986	0.16638	0.1977*	0.419 (6)
H11F	0.69491	−0.07898	0.16696	0.1977*	0.419 (6)
H17	0.43332	0.01407	0.40544	0.0795*	
H16	0.35661	0.08746	0.30122	0.0657*	
H20A	−0.31611	0.01976	0.34291	0.1338*	
H20B	−0.31734	−0.03998	0.41394	0.1338*	
H20C	−0.28119	−0.08168	0.33812	0.1338*	
H21A	0.18963	0.13638	0.14277	0.0977*	
H21B	0.27359	0.06069	0.18276	0.0977*	
H22A	0.01994	0.04238	0.11918	0.1582*	0.581 (6)
H22B	0.08591	−0.03154	0.16917	0.1582*	0.581 (6)
H23A	0.14796	0.01266	0.02539	0.1977*	0.581 (6)
H23B	0.11414	−0.08189	0.05589	0.1977*	0.581 (6)
H23C	0.25133	−0.03867	0.07639	0.1977*	0.581 (6)
H24A	0.25346	0.27095	0.23705	0.1170*	
H24B	0.24125	0.24753	0.32089	0.1170*	
H24C	0.16432	0.32910	0.28629	0.1170*	
H18	0.29179	−0.06454	0.47530	0.0778*	
H19	0.06943	−0.06503	0.44334	0.0684*	
H22C	0.24098	−0.02177	0.10690	0.1582*	0.419 (6)
H22D	0.18982	−0.05415	0.18295	0.1582*	0.419 (6)
H23D	−0.02474	−0.02240	0.14340	0.1977*	0.419 (6)
H23E	0.03615	−0.08499	0.08474	0.1977*	0.419 (6)
H23F	0.02312	0.01742	0.06925	0.1977*	0.419 (6)

### Atomic displacement parameters (Å^2^)

**Table:**

	*U*^11^	*U*^22^	*U*^33^	*U*^12^	*U*^13^	*U*^23^
S1	0.0426 (6)	0.0712 (8)	0.0403 (6)	0.0018 (5)	0.0048 (4)	0.0047 (6)
O1	0.0583 (19)	0.100 (3)	0.088 (3)	−0.0200 (19)	0.0010 (18)	0.031 (2)
O2	0.0456 (17)	0.096 (3)	0.080 (2)	0.0064 (17)	−0.0020 (16)	0.028 (2)
O3	0.0594 (18)	0.098 (3)	0.054 (2)	0.0344 (17)	−0.0012 (15)	−0.0018 (18)
O4	0.0695 (19)	0.106 (3)	0.0490 (19)	−0.0232 (18)	0.0167 (16)	0.0120 (19)
N1	0.0535 (19)	0.056 (2)	0.040 (2)	0.0046 (17)	−0.0007 (16)	0.0079 (17)
C1	0.049 (3)	0.072 (4)	0.042 (3)	−0.005 (2)	0.000 (2)	0.008 (2)
C2	0.048 (2)	0.048 (3)	0.037 (2)	−0.0028 (19)	0.0003 (19)	0.001 (2)
C3	0.061 (3)	0.059 (3)	0.038 (2)	−0.007 (2)	0.001 (2)	0.001 (2)
C4	0.074 (3)	0.064 (3)	0.043 (3)	−0.015 (2)	0.018 (2)	−0.005 (2)
C5	0.051 (3)	0.083 (4)	0.057 (3)	−0.007 (2)	0.018 (2)	−0.008 (3)
C6	0.051 (2)	0.067 (3)	0.049 (3)	0.004 (2)	0.003 (2)	−0.003 (2)
C7	0.048 (2)	0.050 (3)	0.037 (2)	−0.0003 (19)	0.0030 (19)	−0.0008 (19)
C8	0.049 (3)	0.143 (6)	0.099 (5)	0.006 (3)	0.001 (3)	0.022 (4)
C9	0.106 (4)	0.059 (4)	0.072 (4)	0.008 (3)	0.005 (3)	0.012 (3)
C10A	0.156 (6)	0.125 (5)	0.116 (4)	0.008 (4)	0.018 (4)	−0.031 (4)
C11A	0.156 (6)	0.125 (5)	0.116 (4)	0.008 (4)	0.018 (4)	−0.031 (4)
C12	0.063 (3)	0.064 (3)	0.054 (3)	−0.006 (2)	0.000 (2)	0.000 (2)
C11B	0.156 (6)	0.125 (5)	0.116 (4)	0.008 (4)	0.018 (4)	−0.031 (4)
C10B	0.156 (6)	0.125 (5)	0.116 (4)	0.008 (4)	0.018 (4)	−0.031 (4)
S2	0.0460 (6)	0.0661 (8)	0.0621 (8)	0.0074 (5)	0.0061 (5)	0.0158 (6)
O5	0.065 (2)	0.116 (3)	0.062 (2)	−0.028 (2)	−0.0037 (18)	0.012 (2)
O6	0.0513 (18)	0.103 (3)	0.062 (2)	−0.0208 (17)	0.0156 (16)	−0.0030 (19)
O7	0.0662 (19)	0.086 (2)	0.077 (2)	0.0189 (17)	0.0312 (18)	0.0109 (19)
O8	0.076 (2)	0.114 (3)	0.074 (2)	0.023 (2)	−0.0118 (19)	0.035 (2)
N2	0.051 (2)	0.057 (2)	0.041 (2)	0.0002 (17)	0.0104 (16)	0.0067 (17)
C13	0.055 (3)	0.060 (3)	0.052 (3)	−0.016 (2)	0.004 (3)	0.004 (2)
C14	0.048 (2)	0.044 (3)	0.043 (3)	−0.0025 (19)	0.004 (2)	0.002 (2)
C15	0.047 (2)	0.044 (3)	0.044 (3)	0.0004 (19)	0.0072 (19)	−0.001 (2)
C16	0.048 (3)	0.049 (3)	0.068 (3)	0.000 (2)	0.010 (2)	0.006 (2)
C17	0.048 (3)	0.063 (3)	0.086 (4)	0.003 (2)	−0.010 (3)	0.001 (3)
C18	0.078 (3)	0.056 (3)	0.059 (3)	0.007 (3)	−0.013 (3)	0.007 (2)
C19	0.065 (3)	0.054 (3)	0.052 (3)	−0.006 (2)	0.005 (2)	0.006 (2)
C20	0.054 (3)	0.119 (5)	0.096 (4)	−0.029 (3)	0.014 (3)	−0.009 (4)
C21	0.103 (4)	0.089 (4)	0.054 (3)	0.002 (3)	0.017 (3)	0.001 (3)
C22A	0.156 (6)	0.125 (5)	0.116 (4)	0.008 (4)	0.018 (4)	−0.031 (4)
C23A	0.156 (6)	0.125 (5)	0.116 (4)	0.008 (4)	0.018 (4)	−0.031 (4)
C24	0.070 (3)	0.055 (3)	0.108 (4)	−0.002 (2)	0.006 (3)	0.008 (3)
C23B	0.156 (6)	0.125 (5)	0.116 (4)	0.008 (4)	0.018 (4)	−0.031 (4)
C22B	0.156 (6)	0.125 (5)	0.116 (4)	0.008 (4)	0.018 (4)	−0.031 (4)

### Geometric parameters (Å, °)

**Table:**

S1—O3	1.422 (3)	C11A—H11B	0.9600
S1—O4	1.425 (3)	C11A—H11C	0.9600
S1—N1	1.630 (3)	C11A—H11A	0.9600
S1—C12	1.743 (5)	C11B—H11D	0.9600
S2—O7	1.422 (3)	C11B—H11E	0.9600
S2—O8	1.418 (4)	C11B—H11F	0.9600
S2—N2	1.622 (3)	C12—H12C	0.9600
S2—C24	1.739 (5)	C12—H12A	0.9600
O1—C1	1.187 (6)	C12—H12B	0.9600
O2—C1	1.330 (5)	C13—C14	1.485 (6)
O2—C8	1.449 (5)	C14—C15	1.395 (6)
O5—C13	1.199 (6)	C14—C19	1.384 (6)
O6—C13	1.311 (6)	C15—C16	1.376 (6)
O6—C20	1.449 (5)	C16—C17	1.366 (7)
N1—C7	1.431 (5)	C17—C18	1.361 (7)
N1—C9	1.488 (6)	C18—C19	1.382 (7)
N2—C21	1.486 (6)	C21—C22A	1.465 (16)
N2—C15	1.442 (5)	C21—C22B	1.53 (2)
C1—C2	1.492 (6)	C22A—C23A	1.51 (2)
C2—C3	1.393 (5)	C22B—C23B	1.50 (3)
C2—C7	1.383 (6)	C16—H16	0.9300
C3—C4	1.371 (7)	C17—H17	0.9300
C4—C5	1.381 (7)	C18—H18	0.9300
C5—C6	1.370 (7)	C19—H19	0.9300
C6—C7	1.390 (6)	C20—H20A	0.9600
C9—C10A	1.482 (15)	C20—H20B	0.9600
C9—C10B	1.527 (19)	C20—H20C	0.9600
C10A—C11A	1.546 (19)	C21—H21A	0.9700
C10B—C11B	1.48 (2)	C21—H21B	0.9700
C3—H3	0.9300	C22A—H22A	0.9700
C4—H4	0.9300	C22A—H22B	0.9700
C5—H5	0.9300	C22B—H22D	0.9700
C6—H6	0.9300	C22B—H22C	0.9700
C8—H8C	0.9600	C23A—H23B	0.9600
C8—H8A	0.9600	C23A—H23C	0.9600
C8—H8B	0.9600	C23A—H23A	0.9600
C9—H9B	0.9700	C23B—H23D	0.9600
C9—H9A	0.9700	C23B—H23F	0.9600
C10A—H10A	0.9700	C23B—H23E	0.9600
C10A—H10B	0.9700	C24—H24B	0.9600
C10B—H10C	0.9700	C24—H24C	0.9600
C10B—H10D	0.9700	C24—H24A	0.9600
			
O3—S1—O4	119.22 (19)	H11D—C11B—H11F	110.00
O3—S1—N1	107.47 (17)	H11E—C11B—H11F	109.00
O3—S1—C12	107.5 (2)	C10B—C11B—H11D	110.00
O4—S1—N1	107.69 (18)	H11D—C11B—H11E	109.00
O4—S1—C12	107.1 (2)	H12B—C12—H12C	109.00
N1—S1—C12	107.4 (2)	S1—C12—H12A	109.00
O7—S2—O8	119.26 (19)	S1—C12—H12B	109.00
O7—S2—N2	107.31 (18)	S1—C12—H12C	109.00
O7—S2—C24	107.4 (2)	H12A—C12—H12B	109.00
O8—S2—N2	107.3 (2)	H12A—C12—H12C	110.00
O8—S2—C24	108.1 (2)	O5—C13—O6	122.3 (4)
N2—S2—C24	106.9 (2)	O5—C13—C14	125.1 (4)
C1—O2—C8	116.0 (4)	O6—C13—C14	112.4 (4)
C13—O6—C20	116.0 (4)	C13—C14—C15	123.2 (4)
S1—N1—C7	118.7 (3)	C13—C14—C19	118.4 (4)
S1—N1—C9	117.8 (3)	C15—C14—C19	118.3 (4)
C7—N1—C9	117.5 (3)	N2—C15—C14	120.3 (3)
S2—N2—C15	117.2 (3)	N2—C15—C16	119.8 (4)
S2—N2—C21	117.9 (3)	C14—C15—C16	120.0 (4)
C15—N2—C21	117.6 (3)	C15—C16—C17	120.7 (4)
O1—C1—O2	123.3 (4)	C16—C17—C18	120.4 (4)
O1—C1—C2	125.7 (4)	C17—C18—C19	119.8 (5)
O2—C1—C2	110.9 (4)	C14—C19—C18	120.9 (4)
C1—C2—C3	118.3 (3)	N2—C21—C22A	106.0 (7)
C1—C2—C7	122.3 (3)	N2—C21—C22B	117.1 (9)
C3—C2—C7	119.3 (4)	C21—C22A—C23A	116.7 (11)
C2—C3—C4	121.1 (4)	C21—C22B—C23B	109.7 (14)
C3—C4—C5	119.4 (5)	C15—C16—H16	120.00
C4—C5—C6	120.2 (4)	C17—C16—H16	120.00
C5—C6—C7	120.9 (4)	C16—C17—H17	120.00
C2—C7—C6	119.2 (4)	C18—C17—H17	120.00
N1—C7—C6	119.1 (4)	C17—C18—H18	120.00
N1—C7—C2	121.7 (4)	C19—C18—H18	120.00
N1—C9—C10A	120.2 (7)	C14—C19—H19	119.00
N1—C9—C10B	103.5 (8)	C18—C19—H19	120.00
C9—C10A—C11A	100.9 (10)	O6—C20—H20A	110.00
C9—C10B—C11B	123.0 (16)	O6—C20—H20B	109.00
C2—C3—H3	119.00	O6—C20—H20C	109.00
C4—C3—H3	119.00	H20A—C20—H20B	109.00
C3—C4—H4	120.00	H20A—C20—H20C	109.00
C5—C4—H4	120.00	H20B—C20—H20C	109.00
C6—C5—H5	120.00	N2—C21—H21A	111.00
C4—C5—H5	120.00	N2—C21—H21B	111.00
C5—C6—H6	120.00	C22A—C21—H21A	111.00
C7—C6—H6	120.00	C22A—C21—H21B	111.00
O2—C8—H8B	110.00	H21A—C21—H21B	109.00
O2—C8—H8A	109.00	C22B—C21—H21A	126.00
H8B—C8—H8C	109.00	C22B—C21—H21B	78.00
H8A—C8—H8C	109.00	C21—C22A—H22A	108.00
O2—C8—H8C	109.00	C21—C22A—H22B	108.00
H8A—C8—H8B	109.00	C23A—C22A—H22A	108.00
N1—C9—H9A	107.00	C23A—C22A—H22B	108.00
N1—C9—H9B	107.00	H22A—C22A—H22B	107.00
C10A—C9—H9A	107.00	C23B—C22B—H22D	110.00
C10A—C9—H9B	107.00	H22C—C22B—H22D	108.00
C10B—C9—H9A	131.00	C21—C22B—H22C	110.00
C10B—C9—H9B	99.00	C21—C22B—H22D	110.00
H9A—C9—H9B	107.00	C23B—C22B—H22C	110.00
C9—C10A—H10A	112.00	C22A—C23A—H23C	109.00
C11A—C10A—H10A	112.00	H23A—C23A—H23B	110.00
C11A—C10A—H10B	112.00	H23A—C23A—H23C	109.00
C9—C10A—H10B	112.00	C22A—C23A—H23A	109.00
H10A—C10A—H10B	109.00	C22A—C23A—H23B	109.00
C9—C10B—H10C	107.00	H23B—C23A—H23C	109.00
C9—C10B—H10D	107.00	C22B—C23B—H23D	109.00
H10C—C10B—H10D	107.00	C22B—C23B—H23E	109.00
C11B—C10B—H10D	106.00	H23D—C23B—H23F	109.00
C11B—C10B—H10C	107.00	H23E—C23B—H23F	109.00
H11A—C11A—H11C	109.00	C22B—C23B—H23F	109.00
H11B—C11A—H11C	109.00	H23D—C23B—H23E	110.00
C10A—C11A—H11B	109.00	S2—C24—H24C	110.00
C10A—C11A—H11A	109.00	H24B—C24—H24C	110.00
H11A—C11A—H11B	109.00	H24A—C24—H24B	109.00
C10A—C11A—H11C	109.00	H24A—C24—H24C	109.00
C10B—C11B—H11E	109.00	S2—C24—H24A	109.00
C10B—C11B—H11F	110.00	S2—C24—H24B	109.00
			
O3—S1—N1—C7	38.0 (3)	O1—C1—C2—C7	−40.1 (7)
O3—S1—N1—C9	−170.0 (3)	O2—C1—C2—C3	−40.3 (5)
O4—S1—N1—C7	167.6 (3)	C1—C2—C7—C6	176.5 (4)
O4—S1—N1—C9	−40.4 (3)	C1—C2—C3—C4	−174.5 (4)
C12—S1—N1—C7	−77.4 (3)	C7—C2—C3—C4	1.7 (7)
C12—S1—N1—C9	74.6 (3)	C1—C2—C7—N1	−4.3 (7)
C24—S2—N2—C21	−76.9 (4)	C3—C2—C7—N1	179.7 (4)
O8—S2—N2—C15	−171.7 (3)	C3—C2—C7—C6	0.6 (7)
O7—S2—N2—C15	−42.4 (3)	C2—C3—C4—C5	−2.2 (7)
O7—S2—N2—C21	168.1 (3)	C3—C4—C5—C6	0.6 (7)
O8—S2—N2—C21	38.8 (4)	C4—C5—C6—C7	1.6 (7)
C24—S2—N2—C15	72.6 (3)	C5—C6—C7—C2	−2.1 (7)
C8—O2—C1—C2	178.1 (4)	C5—C6—C7—N1	178.7 (4)
C8—O2—C1—O1	1.8 (6)	N1—C9—C10A—C11A	−74.2 (11)
C20—O6—C13—O5	2.2 (7)	O5—C13—C14—C15	35.4 (7)
C20—O6—C13—C14	−173.4 (4)	O5—C13—C14—C19	−140.6 (5)
S1—N1—C7—C6	103.8 (4)	O6—C13—C14—C15	−149.2 (4)
C9—N1—C7—C2	132.5 (4)	O6—C13—C14—C19	34.9 (6)
C9—N1—C7—C6	−48.3 (5)	C13—C14—C15—N2	5.8 (6)
S1—N1—C9—C10A	146.0 (7)	C13—C14—C15—C16	−175.2 (4)
C7—N1—C9—C10A	−61.7 (8)	C19—C14—C15—N2	−178.3 (4)
S1—N1—C7—C2	−75.4 (5)	C19—C14—C15—C16	0.8 (6)
C15—N2—C21—C22A	95.0 (7)	C13—C14—C19—C18	174.2 (4)
S2—N2—C15—C14	83.4 (4)	C15—C14—C19—C18	−2.0 (7)
S2—N2—C15—C16	−95.7 (4)	N2—C15—C16—C17	179.3 (4)
C21—N2—C15—C14	−127.0 (4)	C14—C15—C16—C17	0.2 (7)
C21—N2—C15—C16	53.9 (6)	C15—C16—C17—C18	0.0 (7)
S2—N2—C21—C22A	−115.6 (7)	C16—C17—C18—C19	−1.2 (7)
O1—C1—C2—C3	135.9 (5)	C17—C18—C19—C14	2.2 (7)
O2—C1—C2—C7	143.7 (4)	N2—C21—C22A—C23A	−170.3 (10)

### Hydrogen-bond geometry (Å, °)

**Table:**

Cg1 and Cg2 are the centroids of the C2–C7 and C14–C19 benzene rings, respectively.

**Table:**

*D*—H···*A*	*D*—H	H···*A*	*D*···*A*	*D*—H···*A*
C6—H6···O8^i^	0.93	2.59	3.483 (6)	161
C11A—H11C···O1	0.96	2.57	3.486 (15)	159
C16—H16···O4	0.93	2.51	3.429 (5)	168
C20—H20C···O3^ii^	0.96	2.57	3.172 (6)	121
C21—H21A···O1	0.97	2.55	3.192 (6)	124
C12—H12A···Cg2^iii^	0.96	2.84	3.660 (5)	144
C18—H18···Cg1^iv^	0.93	2.87	3.588 (5)	135

Symmetry codes: (i) *x*+1, *y*, *z*; (ii) −*x*, *y*−1/2, −*z*+1/2; (iii) −*x*+1, *y*+1/2, −*z*+1/2; (iv) −*x*+1, *y*−1/2, −*z*+1/2.
